# 2-[(*E*)-(6-Amino-1,3-dimethyl-2,4-dioxo-1,2,3,4-tetra­hydro­pyrimidin-5-yl)imino­meth­yl]pyridinium bromide

**DOI:** 10.1107/S1600536811033290

**Published:** 2011-08-27

**Authors:** Irvin Booysen, Muhammed Ismail, Thomas Gerber, Eric Hosten, Richard Betz

**Affiliations:** a University of Kwazulu-Natal, School of Chemistry, Private Bag X01, Scottsville 3209, Pietermaritzburg, South Africa; b Nelson Mandela Metropolitan University, Summerstrand Campus, Department of Chemistry, University Way, Summerstrand, PO Box 77000, Port Elizabeth 6031, South Africa

## Abstract

The title compound, C_12_H_14_N_5_O_2_^+^·Br^−^, is the hydro­bromide salt of a Schiff base in which protonation has taken place at the pyridine N atom. This organic cation is essentially planar (r.m.s. of all fitted non-H atoms = 0.0448 Å). In the crystal, N—H⋯Br hydrogen bonds as well as C—H⋯O and C–H⋯Br inter­actions connect the mol­ecules, forming a three-dimensional network.

## Related literature

For the development of radiopharmaceuticals, see: Gerber *et al.* (2011 ▶). For the crystal structure of the neutral organic parent molecule, see: Booysen *et al.* (2011*a* ▶). For the crystal structures of polymorphs of 6-amino-1,3-dimethyl-5-[(*E*-2-(methyl­sulfan­yl)benzyl­idene­amino]pyrimidine-2,4(1*H*,3*H*)-dione, see: Booysen *et al.* (2011**b* ▶,c* ▶). For graph-set analysis of hydrogen bonds, see: Etter *et al.* (1990 ▶); Bernstein *et al.* (1995 ▶).
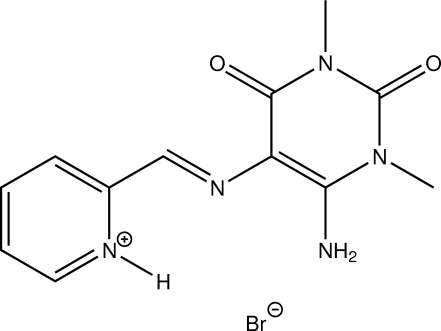


## Experimental

### 

#### Crystal data

C_12_H_14_N_5_O_2_^+^·Br^−^
*M* *_r_* = 340.19
Monoclinic, 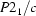

*a* = 8.9520 (2) Å
*b* = 4.9630 (1) Å
*c* = 30.9123 (6) Å
β = 105.391 (1)°
*V* = 1324.14 (5) Å^3^
*Z* = 4
Mo *K*α radiation
μ = 3.11 mm^−1^
*T* = 200 K
0.55 × 0.28 × 0.12 mm

#### Data collection

Bruker APEXII CCD diffractometer
Absorption correction: multi-scan (*SADABS*; Bruker, 2008 ▶) *T*_min_ = 0.660, *T*_max_ = 1.000
10606 measured reflections
3277 independent reflections
2998 reflections with *I* > 2σ(*I*)
*R*_int_ = 0.017

#### Refinement

*R*[*F* ^2^ > 2σ(*F* ^2^)] = 0.029
*wR*(*F* ^2^) = 0.068
*S* = 1.16
3277 reflections
195 parameters
H atoms treated by a mixture of independent and constrained refinement
Δρ_max_ = 0.42 e Å^−3^
Δρ_min_ = −0.36 e Å^−3^

### 

Data collection: *APEX2* (Bruker, 2010 ▶); cell refinement: *SAINT* (Bruker, 2010 ▶); data reduction: *SAINT*; program(s) used to solve structure: *SIR97* (Altomare *et al.*, 1999 ▶); program(s) used to refine structure: *SHELXL97* (Sheldrick, 2008 ▶); molecular graphics: *ORTEP-3* (Farrugia, 1997 ▶) and *Mercury* (Macrae *et al.*, 2008 ▶); software used to prepare material for publication: *SHELXL97* and *PLATON* (Spek, 2009 ▶).

## Supplementary Material

Crystal structure: contains datablock(s) I, global. DOI: 10.1107/S1600536811033290/su2307sup1.cif

Supplementary material file. DOI: 10.1107/S1600536811033290/su2307Isup2.cdx

Structure factors: contains datablock(s) I. DOI: 10.1107/S1600536811033290/su2307Isup3.hkl

Supplementary material file. DOI: 10.1107/S1600536811033290/su2307Isup4.cml

Additional supplementary materials:  crystallographic information; 3D view; checkCIF report

## Figures and Tables

**Table 1:** Hydrogen-bond geometry (Å, °)

*D*—H⋯*A*	*D*—H	H⋯*A*	*D*⋯*A*	*D*—H⋯*A*
N4—H741⋯Br1^i^	0.87 (3)	2.77 (3)	3.4584 (19)	138 (2)
N4—H742⋯Br1^ii^	0.82 (3)	2.55 (3)	3.312 (2)	154 (2)
N5—H751⋯Br1^ii^	0.85 (3)	2.41 (3)	3.1763 (18)	151 (2)
C5—H5*A*⋯Br1^i^	0.98	2.89	3.764 (2)	148
C6—H6*A*⋯O1^iii^	0.98	2.60	3.453 (3)	145
C9—H9⋯O2^iv^	0.95	2.53	3.446 (3)	162
C10—H10⋯O1^v^	0.95	2.56	3.429 (3)	152

Symmetry codes: (i) 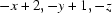; (ii) 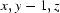; (iii) 
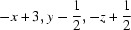
; (iv) 
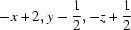
; (v) 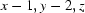.

**Fig. 1.**
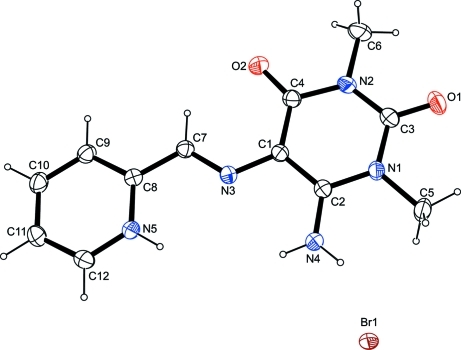
The molecular structure of the title compound, with atom labels and displacement ellipsoids drawn at the 50% probability level.

**Fig. 2.**
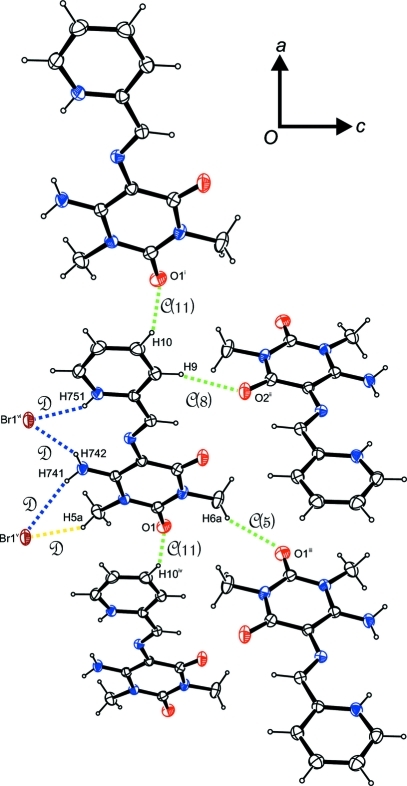
A view along the b-axis of the intermolecular contacts in the crystal of the title compound [Blue dashed lines indicate N-H···Br hydrogen bonds, green dashed lines C–H···O contacts and yellow dashed lines C–H···Br contacts; see Table 1 for details; Symmetry operators: (i) x - 1, y - 2, z; (ii) -x + 2, y - 1/2, -z + 1/2; (iii) -x + 3, y - 1/2,-z + 1/2; (iv) x + 1, y + 2, z; (v) -x + 2, -y + 1, -z; (vi) x, y - 1, z].

**Fig. 3.**
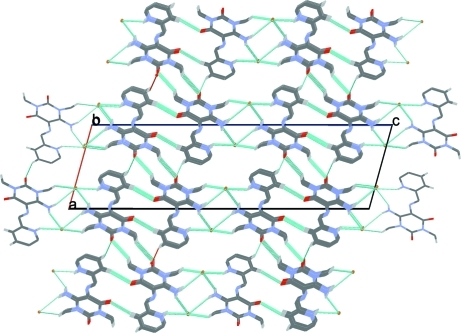
The crystal packing of the title compound, viewed along the b-axis. The N-H···Br, C-H···Br and C-H···O interactions are shown as dashed cyan lines - see Table 1 for details.

## supplementary crystallographic information

### Comment

Next to cardiovascular diseases, cancer has become one of the main fatal diseases in industrialized countries. Apart from classical surgery, chemo- and radiotherapeutic treatments have entered the arsenal of possible cures for certain types of cancer. All methods, however, suffer from their own set of problematic side-effects and, as a consequence, the development of radiopharmaceuticals – combining the advantages of chemotherapy as well as radiation methods while at the same time avoiding their unique respective undesired side-effects – has been a topic of research (Gerber *et al.*, 2011). Tailoring and fine-tuning of the envisioned radiopharmaceuticals' properties such as lipophilicity and, in particular, inertness is of paramount importance with respect to possible future *in vivo* applications in contemporary medicine and requires sound knowledge about structural parameters of the ligands applied if a more heuristic approach in the synthesis is to triumph over pure trial-and-error as it is encountered in this specific field of coordination chemistry up to the present day. To allow for an assessment of changes in structural features upon coordination, the molecular and crystal structure of the title compound has been determined. The crystal structure of the neutral compound (Booysen *et al.*, 2011*a*), and other 6-amino-1,3-dimethyl-2,4(1*H*,3*H*)-dione-derived Schiff-base ligands (Booysen *et al.*, 2011**b*,c*), have been described previously.

The molecular structure of the title molecule is illustrated in Fig. 1. Protonation of the neutral organic ligand took place on the nitrogen atom, N5, of the pyridine moiety. The molecule has the *E* configuration about the C=N bond. As expected the intracyclic angles in the protonated pyridine moiety cover a range of 118.09 (19)–123.33 (19) °, with the largest angle on the protonated nitrogen atom, N, and the smallest angle on the carbon atom, C , bonded to the exocyclic substituent. The organic cation is essentially planar (r.m.s. for all its fitted non-hydrogen atoms = 0.0448 Å).

In the crystal, N-H···Br hydrogen bonds as well as C–H···O and C–H···Br contacts are observed (Table 1). While the hydrogen bonds are formed between the nitrogen-bonded hydrogen atoms and the bromide anion exclusively, the C–H···O contacts involve the hydrogen atoms of the pyridine moiety and one of the nitrogen-bonded methyl groups as donors and both oxygen atoms as acceptors. The C–H···Br contact is supported by one of the hydrogen atoms of the second nitrogen-bonded methyl group. In terms of graph-set analysis (Etter *et al.*, 1990; Bernstein *et al.*, 1995), the descriptor for the classical hydrogen bonds is *DDD* on the unitary level, whereas the C–H···O contacts necessitate a *C*(5)*C*(8)*C*(11) on the same level. In total, these contact result in the formation of a three-dimensional network (Fig. 3).

### Experimental

The title compound was prepared by the reaction of (*E*)-6-amino-1,3- dimethyl-5-(pyridin-2-ylmethyleneamino)pyrimidine-2,4(1*H*,3*H*)- dione and *trans*-[ReOBr_3_(PPh_3_)_2_] in methanol. The solution was filtered and single crystals suitable for the X-ray analysis were obtained from the mother liquor which was left in a fridge for several days.

### Refinement

Carbon-bound H atoms were placed in calculated positions (C—H 0.95 Å) and were included in the refinement in the riding model approximation, with *U*(H) set to 1.2*U*_eq_(C). The H atoms of the methyl groups were allowed to rotate with a fixed angle around the C—C bond to best fit the experimental electron density (HFIX 137 in the *SHELX* program suite (Sheldrick, 2008)), with *U*(H) set to 1.5*U*_eq_(C). All nitrogen-bound H atoms were located on a difference Fourier map and refined freely.

### Crystal data

**Table:**

C_12_H_14_N_5_O_2_^+^·Br^−^	*F*(000) = 688
*M**_r_* = 340.19	*D*_x_ = 1.706 Mg m^−^^3^
Monoclinic, *P*2_1_/*c*	Mo *K*α radiation, λ = 0.71069 Å
Hall symbol: -P 2ybc	Cell parameters from 7045 reflections
*a* = 8.9520 (2) Å	θ = 2.7–28.3°
*b* = 4.9630 (1) Å	µ = 3.11 mm^−^^1^
*c* = 30.9123 (6) Å	*T* = 200 K
β = 105.391 (1)°	Plate, orange
*V* = 1324.14 (5) Å^3^	0.55 × 0.28 × 0.12 mm
*Z* = 4	

### Data collection

**Table:**

Bruker APEXII CCD diffractometer	3277 independent reflections
Radiation source: fine-focus sealed tube	2998 reflections with *I* > 2σ(*I*)
graphite	*R*_int_ = 0.017
φ and ω scans	θ_max_ = 28.3°, θ_min_ = 2.4°
Absorption correction: multi-scan (*SADABS*; Bruker, 2008)	*h* = −11→11
*T*_min_ = 0.660, *T*_max_ = 1.000	*k* = −6→6
10606 measured reflections	*l* = −41→38

### Refinement

**Table:**

Refinement on *F*^2^	Primary atom site location: structure-invariant direct methods
Least-squares matrix: full	Secondary atom site location: difference Fourier map
*R*[*F*^2^ > 2σ(*F*^2^)] = 0.029	Hydrogen site location: inferred from neighbouring sites
*wR*(*F*^2^) = 0.068	H atoms treated by a mixture of independent and constrained refinement
*S* = 1.16	*w* = 1/[σ^2^(*F*_o_^2^) + (0.0154*P*)^2^ + 1.8033*P*] where *P* = (*F*_o_^2^ + 2*F*_c_^2^)/3
3277 reflections	(Δ/σ)_max_ < 0.001
195 parameters	Δρ_max_ = 0.42 e Å^−^^3^
0 restraints	Δρ_min_ = −0.36 e Å^−^^3^

### Fractional atomic coordinates and isotropic or equivalent isotropic displacement parameters (Å^2^)

**Table:**

	*x*	*y*	*z*	*U*_iso_*/*U*_eq_	
O1	1.37652 (19)	0.7382 (4)	0.17215 (6)	0.0318 (4)	
O2	1.1136 (2)	0.0840 (3)	0.22406 (5)	0.0308 (4)	
N1	1.2019 (2)	0.4910 (4)	0.12052 (6)	0.0207 (4)	
N2	1.2433 (2)	0.4126 (4)	0.19775 (6)	0.0231 (4)	
N3	0.94690 (19)	−0.0572 (4)	0.13177 (6)	0.0190 (3)	
N4	1.0173 (2)	0.2538 (4)	0.06812 (6)	0.0242 (4)	
H741	1.053 (3)	0.317 (6)	0.0469 (10)	0.039 (8)*	
H742	0.950 (3)	0.138 (6)	0.0608 (9)	0.026 (7)*	
N5	0.7294 (2)	−0.4378 (4)	0.09421 (6)	0.0222 (4)	
H751	0.763 (3)	−0.333 (6)	0.0774 (9)	0.030 (7)*	
C1	1.0559 (2)	0.1427 (4)	0.14542 (6)	0.0189 (4)	
C2	1.0908 (2)	0.2922 (4)	0.11067 (7)	0.0188 (4)	
C3	1.2809 (2)	0.5581 (4)	0.16435 (7)	0.0226 (4)	
C4	1.1344 (2)	0.2018 (4)	0.19125 (7)	0.0212 (4)	
C5	1.2294 (3)	0.6593 (5)	0.08419 (8)	0.0292 (5)	
H5A	1.2784	0.5504	0.0653	0.044*	
H5B	1.2977	0.8096	0.0971	0.044*	
H5C	1.1304	0.7298	0.0659	0.044*	
C6	1.3189 (3)	0.4895 (6)	0.24431 (8)	0.0354 (6)	
H6A	1.4077	0.3709	0.2564	0.053*	
H6B	1.2448	0.4724	0.2625	0.053*	
H6C	1.3546	0.6766	0.2451	0.053*	
C7	0.8975 (2)	−0.2117 (4)	0.15854 (7)	0.0201 (4)	
H7	0.9368	−0.1947	0.1901	0.024*	
C8	0.7801 (2)	−0.4132 (4)	0.13933 (7)	0.0194 (4)	
C9	0.7160 (2)	−0.5808 (4)	0.16555 (7)	0.0235 (4)	
H9	0.7485	−0.5673	0.1973	0.028*	
C10	0.6044 (3)	−0.7680 (5)	0.14522 (8)	0.0269 (5)	
H10	0.5598	−0.8822	0.1631	0.032*	
C11	0.5580 (3)	−0.7884 (5)	0.09892 (8)	0.0287 (5)	
H11	0.4823	−0.9173	0.0847	0.034*	
C12	0.6233 (2)	−0.6188 (5)	0.07375 (7)	0.0269 (5)	
H12	0.5930	−0.6304	0.0419	0.032*	
Br1	0.75392 (3)	0.85034 (5)	0.004599 (7)	0.03093 (8)	

### Atomic displacement parameters (Å^2^)

**Table:**

	*U*^11^	*U*^22^	*U*^33^	*U*^12^	*U*^13^	*U*^23^
O1	0.0294 (8)	0.0307 (9)	0.0339 (9)	−0.0127 (7)	0.0057 (7)	−0.0055 (7)
O2	0.0388 (9)	0.0325 (9)	0.0189 (7)	−0.0090 (7)	0.0039 (6)	0.0023 (7)
N1	0.0219 (8)	0.0197 (9)	0.0213 (8)	−0.0046 (7)	0.0073 (7)	−0.0011 (7)
N2	0.0253 (9)	0.0225 (9)	0.0190 (8)	−0.0031 (7)	0.0013 (7)	−0.0029 (7)
N3	0.0189 (8)	0.0183 (8)	0.0200 (8)	−0.0004 (7)	0.0054 (6)	−0.0006 (7)
N4	0.0271 (9)	0.0271 (10)	0.0182 (8)	−0.0072 (8)	0.0056 (7)	0.0012 (7)
N5	0.0228 (8)	0.0245 (9)	0.0197 (8)	−0.0040 (7)	0.0067 (7)	0.0021 (7)
C1	0.0192 (9)	0.0187 (10)	0.0186 (9)	−0.0003 (8)	0.0047 (7)	−0.0009 (8)
C2	0.0185 (9)	0.0171 (10)	0.0207 (9)	0.0012 (7)	0.0052 (7)	−0.0008 (8)
C3	0.0193 (9)	0.0227 (10)	0.0250 (10)	0.0000 (8)	0.0042 (8)	−0.0026 (8)
C4	0.0224 (9)	0.0192 (10)	0.0209 (10)	0.0008 (8)	0.0035 (8)	−0.0008 (8)
C5	0.0359 (12)	0.0257 (11)	0.0290 (11)	−0.0091 (10)	0.0138 (9)	0.0017 (10)
C6	0.0413 (13)	0.0355 (14)	0.0223 (11)	−0.0073 (11)	−0.0038 (10)	−0.0053 (10)
C7	0.0211 (9)	0.0205 (10)	0.0188 (9)	−0.0001 (8)	0.0055 (7)	−0.0003 (8)
C8	0.0194 (9)	0.0199 (10)	0.0192 (9)	0.0012 (8)	0.0055 (7)	0.0002 (8)
C9	0.0254 (10)	0.0245 (11)	0.0213 (10)	−0.0004 (9)	0.0075 (8)	0.0024 (8)
C10	0.0245 (10)	0.0271 (11)	0.0301 (11)	−0.0033 (9)	0.0089 (9)	0.0055 (9)
C11	0.0232 (10)	0.0287 (12)	0.0318 (12)	−0.0068 (9)	0.0030 (9)	0.0010 (9)
C12	0.0246 (10)	0.0310 (12)	0.0223 (10)	−0.0043 (9)	0.0015 (8)	−0.0004 (9)
Br1	0.03684 (13)	0.03914 (14)	0.01623 (10)	−0.01255 (11)	0.00606 (8)	−0.00141 (10)

### Geometric parameters (Å, °)

**Table:**

O1—C3	1.217 (3)	C1—C4	1.435 (3)
O2—C4	1.227 (3)	C5—H5A	0.9800
N1—C2	1.377 (3)	C5—H5B	0.9800
N1—C3	1.392 (3)	C5—H5C	0.9800
N1—C5	1.471 (3)	C6—H6A	0.9800
N2—C3	1.373 (3)	C6—H6B	0.9800
N2—C4	1.407 (3)	C6—H6C	0.9800
N2—C6	1.469 (3)	C7—C8	1.458 (3)
N3—C7	1.290 (3)	C7—H7	0.9500
N3—C1	1.377 (3)	C8—C9	1.387 (3)
N4—C2	1.319 (3)	C9—C10	1.387 (3)
N4—H741	0.87 (3)	C9—H9	0.9500
N4—H742	0.82 (3)	C10—C11	1.384 (3)
N5—C12	1.338 (3)	C10—H10	0.9500
N5—C8	1.353 (3)	C11—C12	1.377 (3)
N5—H751	0.85 (3)	C11—H11	0.9500
C1—C2	1.407 (3)	C12—H12	0.9500
			
C2—N1—C3	122.47 (17)	N1—C5—H5C	109.5
C2—N1—C5	119.69 (18)	H5A—C5—H5C	109.5
C3—N1—C5	117.56 (18)	H5B—C5—H5C	109.5
C3—N2—C4	125.60 (18)	N2—C6—H6A	109.5
C3—N2—C6	117.31 (19)	N2—C6—H6B	109.5
C4—N2—C6	117.04 (18)	H6A—C6—H6B	109.5
C7—N3—C1	124.61 (18)	N2—C6—H6C	109.5
C2—N4—H741	121 (2)	H6A—C6—H6C	109.5
C2—N4—H742	120.8 (18)	H6B—C6—H6C	109.5
H741—N4—H742	116 (3)	N3—C7—C8	118.67 (18)
C12—N5—C8	123.33 (19)	N3—C7—H7	120.7
C12—N5—H751	116.5 (18)	C8—C7—H7	120.7
C8—N5—H751	120.1 (18)	N5—C8—C9	118.09 (19)
N3—C1—C2	115.38 (17)	N5—C8—C7	119.33 (18)
N3—C1—C4	124.84 (18)	C9—C8—C7	122.58 (19)
C2—C1—C4	119.77 (18)	C10—C9—C8	119.8 (2)
N4—C2—N1	117.62 (19)	C10—C9—H9	120.1
N4—C2—C1	122.17 (19)	C8—C9—H9	120.1
N1—C2—C1	120.19 (18)	C11—C10—C9	120.0 (2)
O1—C3—N2	122.5 (2)	C11—C10—H10	120.0
O1—C3—N1	121.2 (2)	C9—C10—H10	120.0
N2—C3—N1	116.34 (18)	C12—C11—C10	118.9 (2)
O2—C4—N2	119.16 (19)	C12—C11—H11	120.5
O2—C4—C1	125.2 (2)	C10—C11—H11	120.5
N2—C4—C1	115.60 (18)	N5—C12—C11	119.8 (2)
N1—C5—H5A	109.5	N5—C12—H12	120.1
N1—C5—H5B	109.5	C11—C12—H12	120.1
H5A—C5—H5B	109.5		
			
C7—N3—C1—C2	−178.59 (19)	C6—N2—C4—O2	−3.8 (3)
C7—N3—C1—C4	2.4 (3)	C3—N2—C4—C1	−1.3 (3)
C3—N1—C2—N4	176.60 (19)	C6—N2—C4—C1	176.35 (19)
C5—N1—C2—N4	2.8 (3)	N3—C1—C4—O2	−0.7 (3)
C3—N1—C2—C1	−1.7 (3)	C2—C1—C4—O2	−179.7 (2)
C5—N1—C2—C1	−175.54 (19)	N3—C1—C4—N2	179.18 (18)
N3—C1—C2—N4	3.9 (3)	C2—C1—C4—N2	0.2 (3)
C4—C1—C2—N4	−177.0 (2)	C1—N3—C7—C8	179.27 (18)
N3—C1—C2—N1	−177.84 (17)	C12—N5—C8—C9	−1.5 (3)
C4—C1—C2—N1	1.3 (3)	C12—N5—C8—C7	179.3 (2)
C4—N2—C3—O1	−179.9 (2)	N3—C7—C8—N5	1.8 (3)
C6—N2—C3—O1	2.5 (3)	N3—C7—C8—C9	−177.43 (19)
C4—N2—C3—N1	0.9 (3)	N5—C8—C9—C10	0.6 (3)
C6—N2—C3—N1	−176.75 (19)	C7—C8—C9—C10	179.8 (2)
C2—N1—C3—O1	−178.6 (2)	C8—C9—C10—C11	0.4 (3)
C5—N1—C3—O1	−4.7 (3)	C9—C10—C11—C12	−0.6 (4)
C2—N1—C3—N2	0.7 (3)	C8—N5—C12—C11	1.3 (3)
C5—N1—C3—N2	174.62 (19)	C10—C11—C12—N5	−0.2 (4)
C3—N2—C4—O2	178.6 (2)		

### Hydrogen-bond geometry (Å, °)

**Table:**

*D*—H···*A*	*D*—H	H···*A*	*D*···*A*	*D*—H···*A*
N4—H741···Br1^i^	0.87 (3)	2.77 (3)	3.4584 (19)	138 (2)
N4—H742···Br1^ii^	0.82 (3)	2.55 (3)	3.312 (2)	154 (2)
N5—H751···Br1^ii^	0.85 (3)	2.41 (3)	3.1763 (18)	151 (2)
C5—H5A···Br1^i^	0.98	2.89	3.764 (2)	148.
C6—H6A···O1^iii^	0.98	2.60	3.453 (3)	145.
C9—H9···O2^iv^	0.95	2.53	3.446 (3)	162.
C10—H10···O1^v^	0.95	2.56	3.429 (3)	152.

Symmetry codes: (i) −*x*+2, −*y*+1, −*z*; (ii) *x*, *y*−1, *z*; (iii) −*x*+3, *y*−1/2, −*z*+1/2; (iv) −*x*+2, *y*−1/2, −*z*+1/2; (v) *x*−1, *y*−2, *z*.
